# Pulmonary tuberculosis presenting as henoch–schönlein purpura: Case report and literature review

**DOI:** 10.1097/MD.0000000000022583

**Published:** 2020-10-02

**Authors:** Jie Li, Xiao-Zi Wang, Rui-Cang Wang, Jie Yang, Hong-Ling Hao, Li-Ying Xue

**Affiliations:** a Department of Hematology, Hebei General Hospital; b Department of Pathology, Hebei Medical University, Shijiazhuang, China.

**Keywords:** case report, henoch–schönlein purpura, pulmonary tuberculosis

## Abstract

**Introduction:**

Henoch–Schönlein purpura (HSP) is an extremely rare condition in patients with pulmonary tuberculosis, with only a few reported cases. Compared to patients with typical clinical symptoms, it is difficult to make a definitive diagnosis when HSP presents as an initial manifestation in pulmonary tuberculosis patients. Herein, a case of pulmonary tuberculosis that showed HSP at first was reported, and the related literatures were reviewed.

**Patient Concerns:**

A 24-year-old man presented with palpable purpura on the extremities, accompanied by abdominal pain, bloody stools, and knee pain.

**Diagnoses:**

The patient was diagnosed with pulmonary tuberculosis based on the results of interferon gamma release assays, purified protein derivative test, and computed tomography.

**Interventions:**

The patient was treated with vitamin C and chlorpheniramine for 2 weeks, and the above-mentioned symptoms were relieved. However, 3 weeks later, the purpura recurred with high-grade fever and chest pain during the inspiratory phase. The patient was then treated with anti-tuberculosis drugs, and the purpura as well as the high fever disappeared.

**Outcomes:**

The patient recovered well and remained free of symptoms during the follow-up examination.

**Conclusion:**

Pulmonary tuberculosis presenting with HSP as an initial manifestation is not common. Therefore, it is difficult to clinically diagnose and treat this disease. When an adult patient shows HSP, it is important to consider the possibility of tuberculosis to avoid misdiagnosis and delayed treatment.

## 1 Introduction

Henoch–Schönlein purpura (HSP), a form of vasculitis affecting joints, skin, and other organs, is very common in children, while it is extremely rare in adult patients. HSP is characterized by purpuric rash, arthritis, gastrointestinal, and/or renal involvement. Since the 1990 s, several sets of diagnostic criteria for HSP have been proposed,[^1^,^2^,^3^,^4^] and its diagnosis mainly depends on typical clinical features, signs, and histopathological findings. Many infectious factors, such as viruses and bacteria, have been described as important causative factors for it. However, the manifestation of HSP prior to pulmonary tuberculosis, has rarely been reported.[^5^,^6^] In this study, we report a patient with HSP as an initial manifestation of pulmonary tuberculosis.

## 2 Case report

A 24-year-old male college student was affected with palpable purpura on his extremities, especially on the lower extremities and buttocks. This was accompanied by bloody stools and abdominal and bilateral knee pain. The patient had no chest pain, cough, expectoration, fever, or other recent history of illness. HSP was diagnosed according to the clinical manifestations and examinations. After treatment with vitamin C and chlorpheniramine for 2 weeks, the purpura and knee pain were relieved, and the stools became normal. However, 3 weeks later, the patient had recurrent purpura on the lower extremities and buttocks, with chest pain in the inspiratory phase and high-grade fever (39°C), whereas there was no sputum, chills, or shivering. He was treated with cephalosporin, but the effect was not obvious. The patient was then transferred to our hospital for further examination and therapy.

At our hospital, physical examination showed high fever and abnormal heartbeats (102 beats per minute). Of note, there were obvious palpable purpuric lesions over both legs and buttocks. The patient had no abdominal pain, moist rales of the lungs, or swelling of the knees and ankles. Laboratory examinations, including complete blood count, liver function tests, and renal function tests, were performed, and the main results were shown in Table 1. The results of the complete blood count showed a white blood cell count of 11,620/mm^3^, along with 75.7% neutrophils, 20.6% lymphocytes, and 2.3% monocytes. The hemoglobin level was 15.6 g/dL, and the platelet count was 266,000/mm^3^. The prothrombin time and activated partial thromboplastin time were normal. The C-reactive protein level was 3.33 mg/L, and the erythrocyte sedimentation rate was 16 mm/h. Since the patient had no expectoration, sputum tuberculosis culture could not be performed. However, the patient was double positive for early secretory antigenic target-6 and culture filtrate protein-10 (early secretory antigenic target-6: 44  spots forming cells/2.5 × 10^5^ peripheral blood mononuclear cells; culture filtrate protein-10: 40  spots forming cells /2.5 × 10^5^ peripheral blood mononuclear cells), which indicated that the T-SPOT.TB test, an interferon gamma release assay (IGRA), was positive. Meanwhile, according to the presence of skin nodules larger than 20 mm in diameter and accompanied by blisters, the purified protein derivative (PPD) test showed a positive reaction.

**Table 1:**
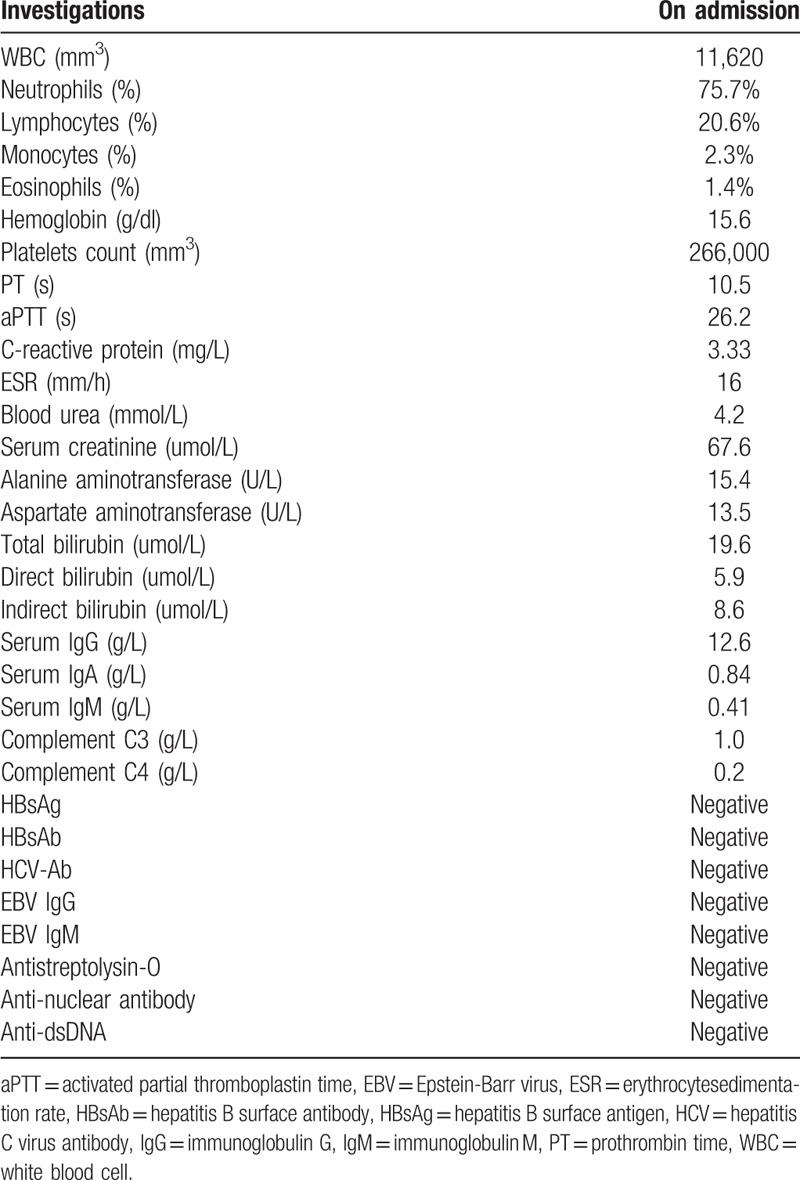
Laboratory investigations.

**Figure 1:**
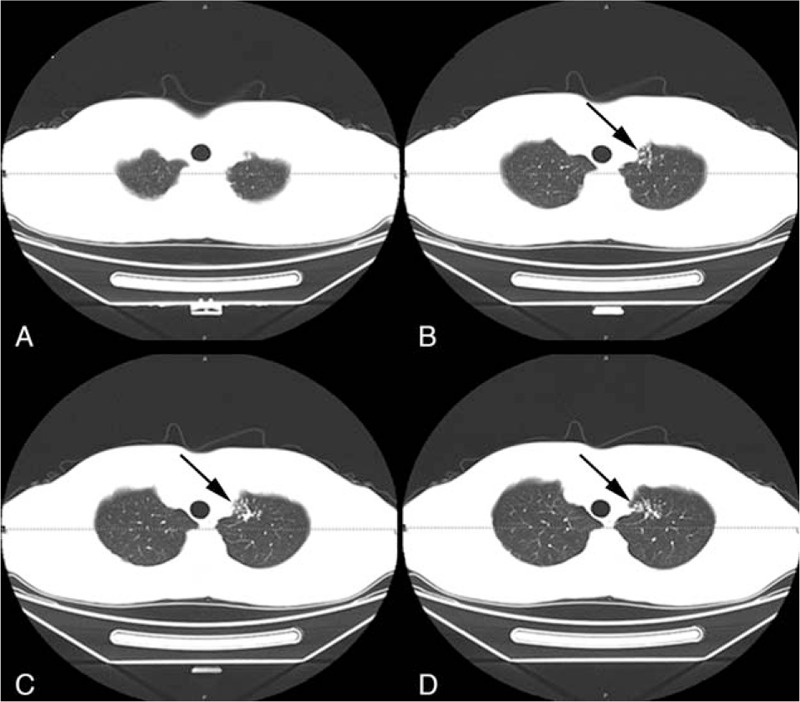
The CT scanning of the lung. (A) There is no real change in the lung field. (B, C, D) CT scanning showed some point patchy shadows in the left apex pulmonis (black arrow). CT = computed tomography.

The patient subsequently underwent an X-ray examination and chest computed tomography (CT) in our hospital. The chest X-ray examination was performed using a Siemens DR Ysio Max scanner (Siemens AG, Munich, Germany). The results showed that both lung fields were well expanded, and lung markings were clear. There was no evidence of lung mass or infiltrate. The bilateral pleural spaces and the mediastinal and bilateral hila were normal. Additionally, chest CT was performed with a Revolution CT ES scanner (GE Medical Systems, LLC, Waukesha, WI), and the scanning showed some patchy shadows in the left apex pulmonis (Fig. 1).

Based on these findings, the patient was diagnosed with pulmonary tuberculosis and consequently received anti-tuberculosis treatment, including a daily dose of isoniazid (300 mg), ethambutol (750 mg), and rifapentine (600 mg). The temperature was normal within 1 week. The chest pain was relieved, and the palpable purpura disappeared gradually after 2 weeks without any obvious unpleasant side effects. No occurrence of HSP, fever, or chest pain was observed after 12 months of follow-up.

Informed consent for publication was obtained from the patient. The study was approved by the Ethics Committee of the Hebei General Hospital.

## 3 Discussion

Tuberculosis is a major public infectious disease worldwide, with enormous challenges in management and diagnosis.^[7]^ The presentation of pulmonary tuberculosis shows a wide variety of hematological manifestations, such as anemia, pancytopenia, myelofibrosis, and thrombocytopenia.[^8^,^9^] However, HSP as an initial manifestation in pulmonary tuberculosis is extremely rare.

HSP is a type of vasculitic syndrome characterized by the eruption of diffuse urticarial plaques and palpable purpura mainly on the lower extremities, abdominal pain, joint pain, and abnormal urinalysis. Besides a mandatory criterion such as palpable purpura (not thrombocytopenia), the latest diagnostic criteria of HSP include at least 1 of the following manifestations: diffuse abdominal pain, leukocytoclastic vasculitis with predominant IgA deposits on skin biopsy, acute arthritis, or arthralgias in any joint, and renal involvement as evidenced by proteinuria and/or hematuria.^[2]^

In the present case, the main symptoms of this patient were palpable purpura with normal platelet count, abdominal pain, bloody stools, and bilateral knee pain without fever, cough, expectoration, and chest pain, as described above. Based on the diagnostic criteria,^[2]^ the patient was diagnosed with HSP. After anti-allergic therapy for 2 weeks, the patient's symptoms, such as purpura and knee pain, subsided. However, 3 weeks later, the purpura recurred, accompanied by high fever, elevated heartbeats, and chest pain during the inspiratory phase. Based on the results of the CT, IGRAs, and PPD test, the patient was diagnosed with pulmonary tuberculosis and had good recovery with anti-tuberculosis treatment. No recurrence of HSP, fever, or chest pain occurred during the follow-up.

In some patients, HSP presents with the classical symptoms of tuberculosis.^[10]^ In other patients, the occurrence of HSP might be induced by factors other than tuberculosis, for example, the anti-tuberculosis agents.^[11]^ We reviewed many studies published in the past 30 years, and 8 adult tuberculosis cases showed HSP as a manifestation. Among these patients, only 3 cases, including our case, presented with HSP before anti-tuberculosis treatment (Table 2). In our case, a 24-year-old male presented with typical symptoms of HSP at first but did not manifest any features of pulmonary tuberculosis, such as low fever and cough. Meanwhile, the patient displayed recurrent purpura after HSP treatment, and the CT scan, PPD test, and IGRAs showed deterministic evidence of tuberculosis. This indicated that HSP must be the initial manifestation of pulmonary tuberculosis in this patient.

**Table 2:**
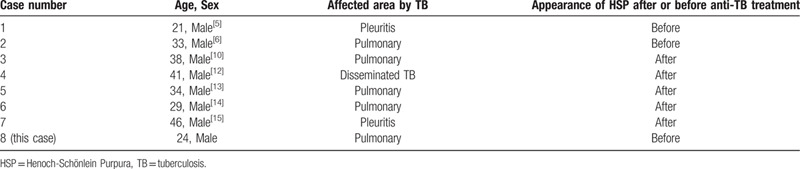
Reported adult cases of hsp in tuberculosis.

A spectrum of infectious agents have close relationships with HSP, such as group A beta-hemolytic streptococcus, *Staphylococcus aureus*, *Helicobacter pylori*, *Hemophilus parainfluenza*, Coxsackie virus, adenovirus, hepatitis A virus, and hepatitis B virus.[^16^,^17^] Nevertheless, very few adult patients with HSP have been reported to be related to *Mycobacterium* tuberculosis. It is supposed that tuberculosis causes vascular damage by invading vessel walls directly or causing immunologic reactions where the immune complexes are deposited on the vessel walls.[^13^,^18^] In most cases, the vascular damage in patients with tuberculosis is mainly caused by immune complexes rather than by direct damage caused by the tubercle bacillus.^[19]^

Furthermore, a hypothesis of HSP pathogenesis indicates that infectious agents may induce the secretion of TGF-β in activated T cells, which then increases the serum IgA levels.^[20]^ Some of these IgA molecules binding to endothelial cells induce endothelial cell lysis by indirectly interacting with the complement, or by directly activating endothelial cells to produce IL-8. As a result, IL-8 activates polymorphonuclear neutrophils to release reactive oxygen metabolites and some proteases, which conversely cause endothelial cell damage.^[20]^ Meanwhile, the serum levels of several inflammatory mediators, such as TNF-α, IL-8, TGF-β, and VEGF, are found to be elevated in patients with HSP.[^21^,^22^,^23^,^24^,^25^,^26^] TNF-α has been reported to increase during the acute stage of HSP and enhance the binding activity of IgA.^[27]^ The latest literature suggests that the human leukocyte antigen region contributes to the genetic risk of tuberculosis,^[28]^ as the major susceptibility locus for IgA vasculitis.^[29]^ IL-10/IL-17 and Th17/Treg cells also play important roles in the pathogenesis of childhood HSP.[^30^,^31^,^32^] To summarize, these theories further suggest that tuberculosis might be an aggravating factor for HSP.

Given the rare case of pulmonary tuberculosis presenting with HSP as an initial manifestation, the main reason for definite diagnosis is the association between pulmonary tuberculosis and HSP. Clinically, the rash occurred repeatedly without definite allergenic or other pathogenic factors, but subsided and did not recur after anti-tuberculosis treatment. Based on these clinical manifestations before and after treatment, it was speculated that there must be a direct correlation between the purpura and tuberculosis. Although this patient did not show symptoms of tuberculosis at first, the CT scan, PPD test, and IGRAs provided deterministic evidence for our conjecture. Multiple studies suggesting that[^20^,^21^,^22^,^23^,^24^,^25^,^26^,^27^,^28^,^29^,^30^,^31^,^32^] the pathogenetic mechanisms of tuberculosis infection could lead to vascular damage through inflammatory mediators further support our diagnosis. However, the pathophysiology of this rare disease is still poorly understood. Therefore, it is still difficult to clinically diagnose and treat this category of patients. Unlike the majority of cases with HSP, our patient was a young adult, and his symptoms were persistent and recurrent. Therefore, when an adult patient shows HSP, it is important to consider the possibility of tuberculosis. Familiar with tuberculosis presentation, early diagnosis and treatment, and good monitoring are necessary to deal with this infectious disease.

## Acknowledgments

We would like to thank Editage (www.editage.com) for English language editing.

## Author contributions

Jie Li, Xiaozi Wang, Ruicang Wang, Jie Yang and Hongling Hao collected and recorded the clinical data. Jie Li and Liying Xue analyzed the case, wrote the manuscript and performed the critical revision.
